# *Lactobacillus iners* and *gasseri*, *Prevotella bivia* and HPV Belong to the Microbiological Signature Negatively Affecting Human Reproduction

**DOI:** 10.3390/microorganisms9010039

**Published:** 2020-12-25

**Authors:** Giuseppina Campisciano, Valerio Iebba, Gabriella Zito, Stefania Luppi, Monica Martinelli, Leo Fischer, Francesco De Seta, Giuseppe Basile, Giuseppe Ricci, Manola Comar

**Affiliations:** 1 Advanced Laboratory of Translational Microbiology, Institute for Maternal and Child Health “IRCCS Burlo Garofolo”, Via dell’Istria 65, 34137 Trieste, Italy; manola.comar@burlo.trieste.it; 2 Department of Medical, Surgical and Health Sciences, University Hospital of Trieste, Strada di Fiume 447, 34149 Trieste, Italy; viebba@units.it (V.I.); francesco.deseta@burlo.trieste.it (F.D.S.); giuseppe.ricci@burlo.trieste.it (G.R.); 3 Obstetrics and Gynecology, Institute for Maternal and Child Health “IRCCS Burlo Garofolo”, Via dell’Istria 65, 34137 Trieste, Italy; gabriella.zito@burlo.trieste.it (G.Z.); stefania.luppi@burlo.trieste.it (S.L.); monica.martinelli@burlo.trieste.it (M.M.); leo.fischertamaro@burlo.trieste.it (L.F.); 4 Orthopedic Department, Clinical Institute San Siro, Via Monreale 18, 20148 Milano, Italy; giuseppe.basile@unife.it

**Keywords:** assisted reproduction, microbiome, bacteriome, virome, infertility

## Abstract

Infertile couples undergoing the use of assisted reproductive technology are a good study model to evaluate the microbiological signatures affecting reproductive health. We tested vaginal lavages, follicular fluids, embryo culture mediums, and seminal fluids from 47 couples for their microbiome composition and HPV infection. Twenty-five infertile couples were diagnosed with unexplained infertility, whereas 22 were diagnosed with explained infertility. Lactobacilli were dominant in the vaginal lavages of both patient groups, and the most abundant species was *L. iners* (CST III), which is linked to a decreased fertility rate. Besides this, *L. gasseri*—which is known to be associated with oocyte DNA fragmentation and decreased sperm mobility—was identified in the seminal fluids, follicular fluids, and embryo culture media of the unexplained infertility group. *Prevotella* was increased in the seminal fluids of the explained infertility group, along with HPV-positive seminal fluids: an infection commonly associated with infertility, especially male infertility. *Prevotella* has been described to negatively affect sperm motility. Taken together, these results suggest that the profiling of the reproductive tract microbiome can add new perspectives to human reproduction.

## 1. Introduction

The impact of the genital tract microbiome on human reproductive physiology is widely studied [1,2,3,4,5]. The disclosure of the role of the female and male urogenital microorganisms in the onset of pregnancy may lead to the identification of new predictive markers of reproductive fitness.

While female commensal genital microorganisms, such as *Lactobacillus* species, may shift the microbial balance of the reproductive environment in favor of pregnancy [6,7], microbial impairments can derive from a biologically-diverse collection of microbes, such as those derived from the sperm microbiome. The most common sperm microbes are the strictly anaerobic *Prevotella* and high proportions of facultative anaerobic bacteria, of which the most abundant bacteria are *Lactobacillus*, *Pseudomonas*, *Gardnerella*, *Finegoldia*, *Corynebacterium*, and *Staphylococcus* [8,9,10]. Although no major shifts in microbiota composition have been observed between fertile and infertile men [8], the presence of a specific bacterial milieu may not be deleterious, but rather necessary for normal sperm function. As an example, an increased relative abundance of *Prevotella* has been observed in samples with defective sperm motility, while an increased relative abundance of *Lactobacillus* has been observed in samples with normal sperm morphology [11]. Besides this, a study from Mandar et al. revealed a strong reciprocal influence exerted by partners’ genital tract microbiota. A significant decrease of the vaginal relative abundance of *L. crispatus* has been described after intercourse, as has the fact that a high amount of *Gardnerella* in the vagina corresponded to higher signs of inflammation of the male genital tract [12,13]. Furthermore, data from couples subject to the use of Assisted Reproductive Technology (ART) show that up to 40% of patients with negative outcomes after in vitro fertilization (IVF) cycles have abnormal microbiota [14,15,16,17].

In addition to bacterial dysbiosis in male and female reproductive tracts, viral infections may affect fertility [18,19]. Among all of them, Human Papilloma Virus (HPV) is the most common sexually-transmitted infection among men and women of reproductive age worldwide, being significantly associated with many adverse effects in reproductive function [20]. In men, HPV can alter sperm motility, is frequently observed in idiopathic infertility, and can increase sperm DNA fragmentation [21,22,23]. In women, HPV infection is associated with a significantly low pregnancy rate compared with women without the infection [24]. A less-studied aspect is the role of HPV in the outcome of assisted reproduction, whereas bacterial dysbiosis is more studied. HPV infection and persistence may be favored by dysbiotic reproductive tract microbiota [25].

The increase of infertility worldwide goes hand in hand with the increased demand for ART. For this reason, we wanted to focus on the possible differences in the genital microbiome and HPV presence in couples diagnosed with unexplained infertility compared with couples diagnosed with explained infertility. We assessed the microbial composition of vaginal lavage, seminal fluid, follicular fluid and embryo culture media from these infertile couples attending ART. Indeed, the addition of new microbiological perspectives may deepen the knowledge of the different infectious infertility causes, especially in unexplained infertility in which no clinical and subclinical abnormalities are detected.

## 2. Materials and Methods

### 2.1. Patient Enrolment

From March to September 2017, 47 couples (mean age 38, range 28–44, Caucasians) attending the Infertility Clinic at the IRCCS Burlo Garofolo Hospital, Trieste, Italy, were included in the study. All of the participants were informed and signed written consent. The study was approved by the ethics committee of the hospital (RC 26/13) and all of the experiments were conducted according to the principles stated in the Declaration of Helsinki. After the finalization of the conventional diagnostic assessment, the couples were classified by the treating physicians into the following groups: idiopathic or unexplained infertility, and explained infertility (e.g., female infertility, male infertility, and couple infertility). During the transvaginal oocyte pick-up, the follicular fluids were obtained by trained gynecologists, avoiding the contamination from the lower genital tract microbiome. Before the embryo implant, the cervical–vaginal lavages were obtained by the injection of 20 mL sterile physiologic solution. Furthermore, the embryo culture media were saved. The seminal fluids used for the in vitro fertilization were obtained from the men by masturbation. The exclusion criteria were the use of vaginal douching, positivity for sexually transmitted diseases (*Chlamydia trachomatis*, *Neisseria gonorrhoeae* and *Trichomonas vaginalis*), and antibiotic/probiotic therapy within 6 months prior to the sample collection. All of the samples were stored at −20 °C until the sample processing.

### 2.2. Nucleic Acid Extraction and Library Preparation

The seminal fluids were pretreated with an aqueous solution of 0.01% Dithiothreitol (DTT), according to the manufacturer’s instruction, starting from 500 µL of the sample. The cervical–vaginal lavages and the follicular fluids were centrifuged at 5000× *g* for 20 min, then the supernatants were discarded, except for 2 mL. For the extraction, 500 µL of the samples were used. The embryo culture media were used in toto (100 µL) for the extraction. The final elution volumes for all of the samples was 50 µL. The extractions were performed using the NucliSENS^®^ easyMAG^®^ system (BioMèrieux, Gorman, NC, USA), according to manufacturer’s instructions.

In order to characterize the composition and structure of the bacterial communities, we sequenced the V3 region of the 16S rRNA gene. The amplicons were obtained by a real-time EvaGreen PCR (EvaGreen^®^ dye, Fisher Molecular Biology, Waltham, MA, USA) using the degenerate primer 27FYM (5′-AGR GTT YGA TYM TGG CTC AG-3′) and the primer U534R, targeting the V1–V3 region (500 bp) in order to allow for the construction of rich libraries. A nested PCR, targeting the V3 region (200 bp), was performed with the primers B338F_P1-adaptor (B338F 5′-ACTCCTACGGGAGGCAGC-3′) and U534R_A_barcode (U534R 5′-ATTACCGCGGCTGCTGG-3′). Each PCR reaction (sample) contained a unique IonXpress Barcode Adapter attached to the reverse primer. No-template controls were processed with the clinical samples. The PCR reactions were performed using the Kapa 2G HiFi Hotstart ready mix 2× (Kapa Biosystems, Waltham, MA, USA), which has the robust amplification that is necessary for NGS (Next Generation Sequencing), and 400 ng/µL BSA. The amplification of the fragments was accomplished using an initial denaturation step of 95 °C for 5 min, followed by a maximum of 25 cycles for the V1–V3 PCR and a maximum of 13 cycles for the V3 PCR of a denaturation step at 95 °C for 30 s, annealing at 59 °C (V1-V3 PCR)/57 °C (V3 PCR) for 30 s, and extension at 72 °C for 45 s. A final extension step of 10 min at 72 °C was performed. The concentrations of the amplicons were estimated using a Qubit^®^ 2.0 Fluorometer (Invitrogen, Carlsbad, CA, USA), and roughly equal amounts (~50 ng) of all of the amplicons were mixed in a single tube and diluted to a concentration of 100 pM. The template preparation was performed using the Ion PGM Hi-Q View kit on the Ion OneTouch™ 2 System (Life Technologies, Grand Island, New York, NY, USA), and was sequenced using the Ion PGM Hi-Q View sequencing kit (Life Technologies, New York, NY, USA) with the Ion PGM™ System technology.

### 2.3. HPV Detection

The vaginal lavages and seminal fluids were tested for HPV DNA. The primer set MY09/MY11 was used, with an amplicon size of 150 bp [26]. The thermal cycling conditions were set as follows: a denaturation step of 95 °C for 9 min, followed by 40 cycles of a denaturation step at 95 °C for 1 min, annealing at 55 °C for 1 min, and extension at 72 °C for 1 min. A final extension step of 7 min at 72 °C was performed. As a control for the DNA extraction, Beta globin was tested in each sample, using the primer PC03/PC04, with an amplicon size of 110 bp. The thermal cycling conditions were set as follows: a denaturation step of 94 °C for 5 min, followed by 40 cycles of a denaturation step at 94 °C for 30 s, annealing at 51 °C for 30 s, and extension at 72 °C for 30 s. A final extension step of 5 min at 72 °C was performed [27]. Positive and negative controls were added. A 5% polyacrylamide gel was used to assess the presence of the amplicon.

### 2.4. Next Generation Sequencing Data Processing

The FastQ files were processed using QIIME 2.0, version 2020.2 [28,29], retaining reads with Q ≥ 20 and read length 175 bp, after DADA2 denoising. Any sequence was removed if it had ambiguous bases or a homopolymer length > 8. In order to keep a consistent classification on the genus and species level, we used a reference taxonomy specifically produced for vaginal microbiota [30] with a BLAST+ consensus. Further analysis was carried out on a random subset of 10,000 reads/sample, using a similarity threshold of 97%. The diversity analyses were performed according to Chao1 and Shannon (alpha diversity) metrics, and according to weighted and unweighted UniFrac distance (beta diversity) metrics.

### 2.5. Statistical Analysis

The variables in dataset were normalized (brute force) and standardized before any statistical analysis by means of Scikit-learn v0.23.2, in order to: (i) homogenize the different dynamic ranges inherently present within the dataset; (ii) ensure a proper comparison at each taxonomical level. The differences in the alpha diversity were assessed by the Kruskal–Wallis test, while for the beta diversity comparisons, the PERMANOVA (Permutational Analysis of Variance) test was used. In order to identify the biomarkers explaining the differences between the groups, we used the linear discriminant analysis (LDA) effect size (LEfSe) method. A t-test was used for the pairwise comparisons on the normalized and standardized dataset. A two-stage False Detection Rate (FDR) of 10% was performed for both the t-test and the non-parametric tests.

### 2.6. Data Availability

The dataset has been deposited in the NCBI Sequence Read Archive (SRA) under the accession number PRJNA480201.

## 3. Results

### 3.1. Features of the Study Cohort

This observational prospective study included 47 consecutive infertile couples (mean age 38, range 28–44) who, from March to September 2017, attended the Infertility Clinic at the IRCCS Burlo Garofolo Hospital, Trieste, Italy. After the finalization of the conventional diagnostic assessment, the couples were classified by the treating physicians into the following groups: twenty-five couples were diagnosed with idiopathic infertility, whereas twenty-two couples were diagnosed with explained infertility.

We performed the sequencing of the V3 region of the16S rRNA gene from 188 biological samples, including the cervical–vaginal lavage, the follicular fluid, the seminal fluid, and the embryo culture medium (which is a merged sample from the sequential embryo culture media) from the infertile couples. Seven embryo culture media were not available, and two out of the seven no-template controls analysed did not produce any sequencing output. The sequencing of the remaining samples produced a total of 7,413,839 reads (Q score > 20), and 4511 OTUs (Operational Taxonomic Units) were observed.

### 3.2. HPV Testing

**Figure 1:**
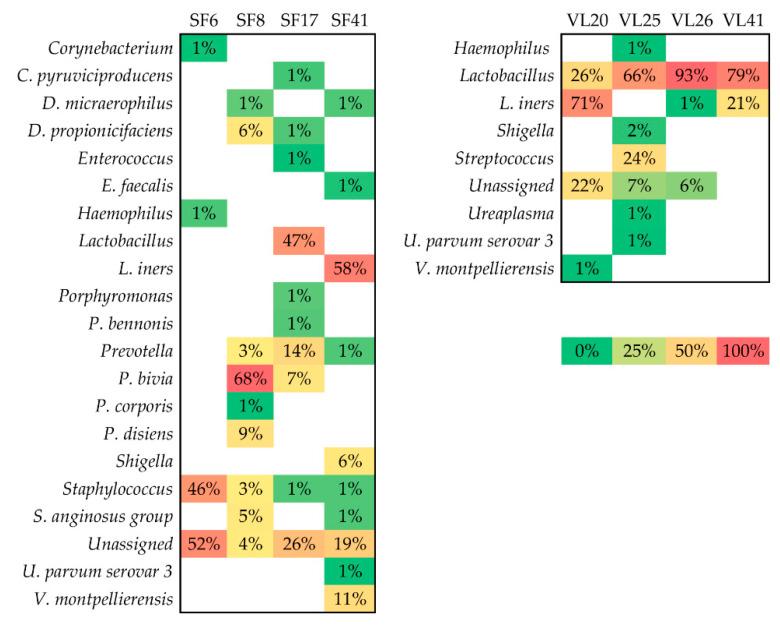
Microbial profile of HPV-positive seminal fluids (SF) and vaginal lavages (VL); the relative abundances of the bacteria identified in HPV-positive samples.

Four seminal fluids and four vaginal lavages tested positive for HPV DNA. Only in one case did the vaginal lavage and seminal fluid belong to the same infertile couple. When comparing HPV-positive samples with HPV-negative samples, *Prevotella* significantly differed (FDR *p*-value < 0.001). In our cohort, *Prevotella* spp. was identified in three out of four HPV-positive seminal fluids (Figure 1). The mean relative abundance of *Prevotella* in HPV-positive samples was 25%, while in the HPV-negative samples, it was 17%.

### 3.3. Taxonomic Biomarkers

In order to eliminate from the analysis the sequencing contaminants, we calculated the core microbiome of the negative controls. The species shared by half of the no-template controls were *Corynebacterium felinum*, *Delftia acidovorans*, *Microbacterium mitrae*, *Chryseobacterium gleum*, *Hyphomicrobium methylovorum*, *Roseateles depolymerans*, *Aureimonas altamirensis*, *Methylophilus leisingeri*, *Pseudomonas aeruginosa*, and *Pseudomonas gessardii*, while the species shared by all of the no-template controls were *Delftia acidovorans* and *Pseudomonas aeruginosa*. For the following analyses, these species were not considered relevant when they were identified in the biological samples at a lower percentage than in the no-template controls.

In order to proceed with the statistical analysis, we rarefied the feature table at a depth of 10,000 sequences/sample. After having rarefied the feature table, we tested for the microbial differences among the groups.

According to the alpha diversity metrics (Chao1 and Shannon), there were no significant differences between the samples from the unexplained infertility group and those from the explained infertility group (Table 1).

According to the weighted and unweighted Bray–Curtis distance (beta diversity) metrics, there were no significant differences when comparing the samples from the explained infertility to those of the unexplained infertility group.

In order to find out which microbes most likely explained the differences between the groups, we applied the LEfSe (Linear discriminant analysis Effect Size) procedure, using the rarefied feature table as the input.

Specific bacterial genera, namely, *Lactobacillus*, *Porphyromonas*, *Prevotella*, *Staphyloccocus*, *Streptococcus*, and the *Streptococcus anginosus* group differed in the comparison of the samples from the unexplained infertility group with those of the explained infertility group (Table 2).

At the species level, *Porphyromonas bennonis* and *Prevotella bivia/disiens* were identified as biomarkers. The results of the LEfSE test are shown in Table 3.

**Figure 2:**
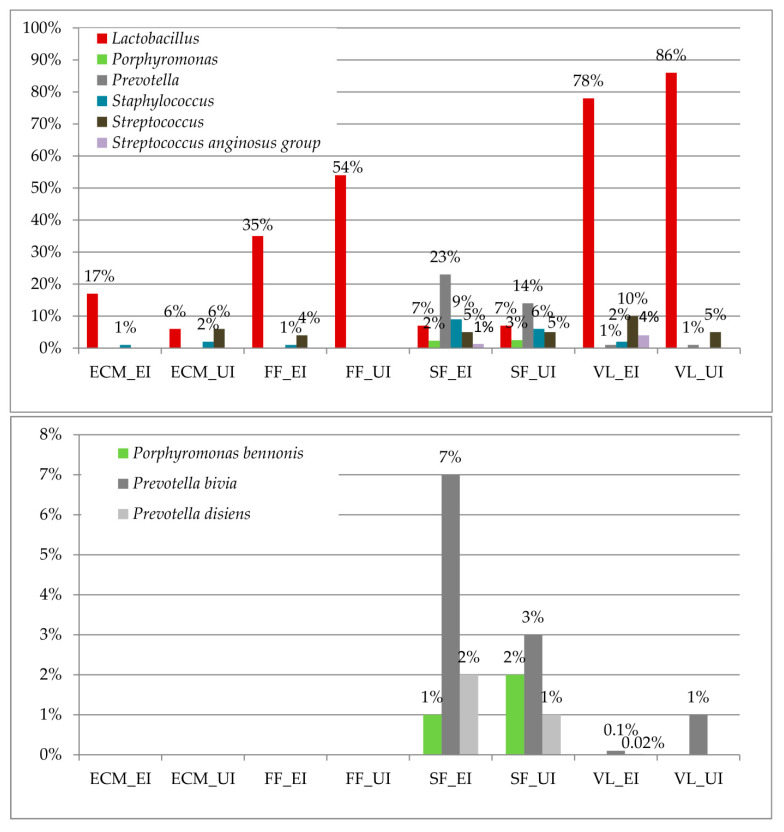
Relative abundances of biomarkers at the genus and species bacterial level; the relative abundances of the bacterial genera/species identified as biomarkers by the LEfSe test in the biological samples (ECM = embryo culture media; FF = follicular fluids; SF = seminal fluids; VL = vaginal lavages; UI = unexplained infertility; EI = explained infertility).

Figure 2 shows the relative abundance of significantly different genera and bacterial species. The relative abundance of lactobacilli was increased in the follicular fluids (54%) and vaginal lavages (86%) of the unexplained infertility group. In the explained infertility group, the seminal fluids showed the increase of *Prevotella*, especially *p. bivia*, and *Staphylococcus*.

**Figure 3:**
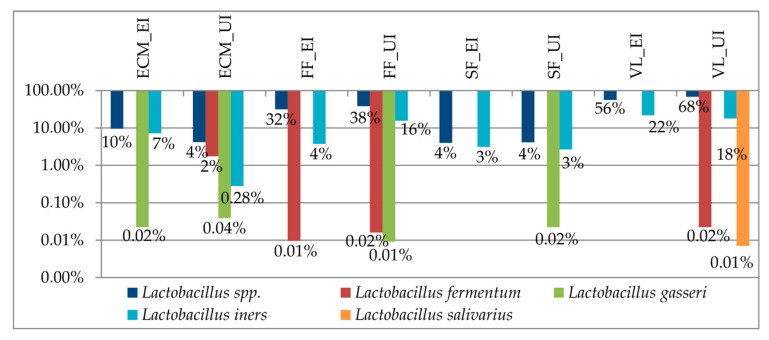
Relative abundances of the *Lactobacillus* species in the study cohort. Abbreviations: ECM = embryo culture media; FF = follicular fluids; SF = seminal fluids; VL = vaginal lavages; UI = unexplained infertility; EI = explained infertility).

Among the *Lactobacillus* species, the predominant species identified in the study cohort was *L. iners*, which corresponds to the vaginal community state type (CST) III [31]. A low amount of *L. gasseri* was also identified, corresponding to the CST II. The *L. iners* amount significantly (FDR *p* value = 0.04) differed among the samples (Figure 3).

## 4. Discussion

The genital tract microbiome of women and men influences the physiology of human reproduction, revealing that a ‘microbiological homeostasis’ is needed for pregnancy establishment [32].

In this regard, a key point is how a resident microbiome can take part in the favorable environment needed for pregnancy, and how it can counteract the action of detrimental factors, such as sexually transmitted infections (STIs). Among these, HPV deserves particular attention, as it is linked to infertility and is the most common STI, to such an extent that—at least once in their life—sexually active men and women will be infected [33,34]. In order to evaluate whether specific microbiological signatures correlate with the fertility status, a good study model is individuated in the clinically-infertile couples undergoing the use of assisted reproductive technology (ART).

Data from our study highlighted a different microbial composition between the genital tracts of couples diagnosed with unexplained infertility and those of couples diagnosed with explained infertility. An overlap between the bacterial composition of the seminal fluids and vaginal lavages of the explained infertility group has been observed. In particular, *Staphylococcus*, *Streptococcus*, and the *Streptococcus anginosus* group were shared among these samples (Figure 2). In accordance with the scientific literature, previous studies have revealed that the isolation of *Enterococci*, *Enterobacteriaceae*, *Streptococci*, *Staphylococci*, and/or Gram-negative bacteria from vaginal lavages is correlated with a lower implantation rate, a decreased number of at-term pregnancies, and an increased number of miscarriages [15,35]. As a discrete amount, these bacteria constitute a physiological vaginal microbiome; it is possible to speculate that, if they were overgrown because of a vaginal dysbiosis or because they were derived from sperm, these bacteria could create a dysbiotic adverse environment against the onset of pregnancy [31].

Another aspect concerns the amount of lactobacilli in the vaginal niche. In comparison with the unexplained group, the vaginal lavages of the explained infertility group showed a decrease of lactobacilli (Figure 2). Among the identified species, a higher abundance of *L. iners* was detected (Figure 3). As shown in previous studies, *L. iners* is indicative of a transitional stage between an abnormal and normal vaginal microbiome because of treatment, or because of the artificially high estrogen levels that occur during IVF [36,37]. We can speculate that the presence of this bacterium constitutes an unfavorable factor for pregnancy establishment.

Concerning the unexplained infertility group, there was a different microbial composition between the seminal fluids and the vaginal lavages, with the seminal fluids showing a higher alpha diversity. There were only some species present in both biological samples, such as lactobacilli and *Streptococcus* (Figure 2). Among the lactobacilli, *L. iners* was also predominant in the vaginal lavages of this group, as seen in the explained infertility group. Conversely, *L. gasseri* was identified at a higher amount in the embryo culture media, follicular fluids, and seminal fluids of the unexplained infertility group compared with the explained infertility group. *L. gasseri* in follicular fluids has been associated with DNA fragmentation in mouse unfertilized oocytes, and—in our cohort—was also identified within the embryo culture media [38]. The identified bacteria within the follicular fluids and embryo culture media overlapped, in part, with the vaginal microbiome, supporting the concept that the bacterial colonization of the upper female genital tract could be a consequence of prior infertility treatment procedures, with bacteria being introduced into the ovary at the time of oocyte retrieval, or could depend on haematogenous bacterial dissemination, as is favored by the significant increase in the ovarian blood supply from the proliferative period of one menstrual cycle to the proliferative phase of the following cycle [1,39]. Besides this, it has been demonstrated that *L. gasseri* significantly reduces sperm motility [40] and that, as a whole, the seminal microbiome used for IVF impacts the embryo quality and pregnancy rates [41]. Indeed, the male factor is considered as a potential predictor of the IVF’s outcome [42,43].

When considering the presence of HPV DNA, four seminal fluids and four vaginal lavages tested positive. These samples, compared with HPV-negative samples, showed a significant increase of *Prevotella* (FDR *p* value < 0.001). In our cohort, *Prevotella* was identified in three out of the four HPV-positive seminal fluids, and with a higher mean relative abundance (25%) compared with HPV-negative seminal fluids (17%). HPV infection is commonly associated with infertility, especially male infertility [33]. Notably, in the vaginal lavages of the unexplained infertility group, the only identified *Prevotella* species was *P. bivia*, whereas, in the vaginal lavages of explained infertility group, several *Prevotella* species were identified (Figure 2). As described in previous studies, *P. bivia* produces high LPS concentrations, which may create a toxic vaginal environment [44]. As for *Staphylococcus* and *Streptococcus*, *Prevotella* is a commensal vaginal bacterium that, if it becomes overgrown, creates a dysbiotic vaginal microbiome [45], which likely affects the fertility status. Besides this, compared with unexplained infertility group, *P. bivia* was increased in the seminal fluids of the explained infertility group, where it has been seen to negatively affect sperm mobility [11].

## 5. Conclusions

Taken together, our results support the concept that the assessment of the reproductive tract microbiome adds a new microbiological perspective to human reproduction. Male and female genital tracts show peculiar microbiomes that can impair the fertility rate.

The seminal microbiome used for IVF needs to be taken into consideration, especially in the diagnosis of unexplained infertility. In this group, the alteration of sperm microbiome, with the increased amount of *Prevotella*, which affects sperm quality, and/or the presence of viral infection, such as HPV, may have a detrimental effect on human reproduction.

On the other hand, another aspect to be taken into consideration, regardless of the infertility diagnosis, is the characterization of the *Lactobacillus* species present in the vaginal lavages. More precisely, *L. iners* and *L. gasseri* can negatively impact the fertility rate. Besides this, the characterization of the microbiome of follicular fluids and embryo culture media may be considered as an adjunctive test when no alterations of the sperm/vaginal microbiome have been identified.

## Figures and Tables

**Table 1:** Alpha diversity. The bacterial diversity values are given as the mean and the 95% confidence interval (CI). All of the pairwise comparisons were performed using a Kruskal–Wallis test (*p* < 0.001). ECM: embryo culture medium.

	CHAO1		
	Explained Infertility	Unexplained Infertility	*p* Value
Vaginal lavages	42 (95% CI = 36–48)	42 (95% CI = 35–49)	0.4
Follicular Fluids	59 (95% CI = 53–65)	63 (95% CI = 49–77)	0.3
Seminal Fluids	94 (95% CI = 79–109)	130 (95% CI = 101–159)	0.08
ECM	24 (95% CI = 19–29)	38 (95% CI = 27–49)	0.1
	**SHANNON**		
	**Explained Infertility**	**Unexplained Infertility**	***p* Value**
Vaginal lavages	1 (95% CI = 0.7–1.3)	1.4 (95% CI = 1.1–1.7)	0.1
Follicular Fluids	2.6 (95% CI = 2.2–3)	2.6 (95% CI = 2.2–3)	0.7
Seminal Fluids	3.7 (95% CI = 3.4–4)	4 (95% CI = 3.7–4.3)	0.09
ECM	2.7 (95% CI = 2.4–3)	3 (95% CI = 2.7–3.3)	0.4

**Table 2:** Biomarkers at the bacterial genus level. The linear discriminative analysis (LDA) effect size (LEfSe) test shows that the bacterial genera were significantly different in the comparison of the samples from the unexplained infertility group with those of the explained infertility group. The relative abundances of the bacterial genera are shown in Figure 2. Positive values of LDA scores (log 10) are indicative of enriched taxa in a given group.

Genus	LDA Score	*p* Value
*Lactobacillus*	6.6	<0.001
*Porphyromonas*	5.09	0.01
*Prevotella*	6.08	<0.001
*Staphylococcus*	5.67	0.05
*Streptococcus*	5.74	0.01
*Streptococcus anginosus* group	5.27	<0.001

**Table 3:** Biomarkers at the bacterial species level. The linear discriminative analysis (LDA) effect size (LEfSe) test shows that the bacterial species were significantly different in the comparison of the samples from the unexplained infertility group with those of the explained infertility group. The relative abundances of the bacterial species are shown in Figure 2. Positive values of LDA scores (log 10) are indicative of enriched taxa in a given group.

Species	LDA Score	*p* Value
*Porphyromonas bennonis*	5.06	0.003
*Prevotella bivia*	5.6	<0.001
*Prevotella disiens*	4.93	<0.001

## Data Availability

The data presented in this study are openly available in the NCBI Sequence Read Archive (SRA) under the accession number PRJNA480201.
